# Visual mismatch negativity in the dorsal stream is independent of concurrent visual task difficulty

**DOI:** 10.3389/fnhum.2013.00411

**Published:** 2013-07-30

**Authors:** Jan Kremláček, Miroslav Kuba, Zuzana Kubová, Jana Langrová, Jana Szanyi, František Vít, Michal Bednář

**Affiliations:** ^1^Department of Pathological Physiology, Faculty of Medicine, Charles University in PragueHradec Králové, Czech Republic; ^2^Department of Rehabilitation, Faculty of Medicine, Charles University in PragueHradec Králové, Czech Republic

**Keywords:** visual mismatch negativity, visual motion, magnocellular pathway, dorsal stream, attention, irrelevant stimulus processing

## Abstract

The manipulation of attention can produce mismatch negativity-like components that are not necessarily connected to the unintentional sensory registration of the violation of probability-based regularity. For clinical purposes, attentional bias should be quantified because it can vary substantially among subjects and can decrease the specificity of the examination. This experiment targets the role of attention in the generation of visual mismatch negativity (vMMN). The visual regularity was generated by a sequence of two radial motions while subjects focused on visual tasks in the central part of the display. Attentional load was systematically varied and had three levels, no-load, easy, and difficult. Rare, deviant, and frequent standard motions were presented with a 10/60 ratio in oddball sequences. Data from 12 subjects was recorded from 64 channels and processed. vMMN was identified within the interval of 142–198 ms. The mean amplitude was evaluated during the aforementioned interval in the parietal and fronto-central regions. A general linear model for repeated measures was applied to the mean amplitude with a three-factor design and showed a significant difference [*F*_(1, 11)_ = 17.40, *p* = 0.002] between standard and deviant stimuli and between regions [*F*_(1, 11)_ = 8.40, *p* = 0.01]; however, no significant effect of the task [*F*_(2, 22)_ = 1.26, *p* = 0.30] was observed. The unintentional detection of irregularity during the processing of the visual motion was independent of the attentional load associated with handling the central visual task. The experiment did not demonstrate an effect of attentional load manipulation on mismatch negativity (MMN) induced by the motion-sequence, which supports the clinical utility of this examination. However, used stimulation paradigm should be further optimized to generate mismatch negativity that is stable enough to be usable not only for group comparisons but also for a single subject assessment.

## Introduction

A specific component of the event-related potential (ERP), called Mismatch Negativity (MMN), denotes an electrophysiological correlate of the brain's detection of an unintentional disruption in the regularity of temporal events. The underlying mechanism is currently attributed to the conflict (error) between sensory input and a prediction and is involved in the processes of perceptual learning (Garrido et al., 2009). Originally, the MMN was described in the auditory modality (Naatanen et al., 1978) as a sensory intelligence within the primary sensory cortex that registers deviant events in a series of standard events (Naatanen et al., 2001). Recent studies on this topic identified an analogous response in the visual modality (vMMN) (Pazo-Alvarez et al., 2003).

Similar to the MMN in the auditory modality, utilizing the vMMN may represent a promising approach for the study of implicit perceptual learning in neuropsychiatric patients, as it is an inexpensive and non-invasive method. This method has previously generated positive results in patients with diseases such as Alzheimer disease (Tales and Butler, 2006; Tales et al., 2008), schizophrenia (Urban et al., 2008), depression (Chang et al., 2011), and autism (Cléry et al., 2013) or in abusers of methamphetamine (Hosak et al., 2008; Kremlacek et al., 2008).

Initially the MMN was recognized as a component independent of attention [in the auditory modality it can be elicited during coma or sleep—see (Näätänen et al., 2011)] and is different from the neuronal fatigue response [i.e., it can be elicited in response to an omitted stimulus (Czigler et al., 2006)]. Genuine MMN reflects a biologically important mechanism for the detection of irregularities in the environment (Czigler et al., 2007).

The MMN, as an electrophysiological marker of specific sensory discrimination, can be confounded by concurrent processes that mimic its appearance. One such process is the aforementioned neural fatigue response (refractoriness), during which a neural population of cells shows repetition-induced suppression of responses to standard stimuli, while another neural population of cells responds to different features of the deviant stimulus without suppression. Attention-related negative components can also confound processes (Czigler, 2007) that are connected to the MMN, as attention can change the ERP response in early visual processing without sensory discrimination (Luck et al., 2000)^1^. For this reason a vMMN review (Czigler, 2007) addressed the issue of attention and noted the necessity to control for this potentially confounding effect.

Because the measurement of the vMMN has to control for refractoriness and attention bias, the procedure is typically long and is paired with a demanding task; thus, its clinical utility is limited as the attentional resources of neuro-psychiatric patients are restricted.

Visual processing is initially anatomically separated into three pathways (parvo-, magno- and konio-cellular). It is generally accepted that the parvocellular (sustained) system conducts information about form and color to the ventral stream and that the second magnocellular (transient) system predominantly carries motion information to the dorsal stream (Ungerleider and Mishkin, 1982; Livingstone and Hubel, 1988). Although, in the later stages of processing, the separate inputs are heavily interconnected it is possible to some extent separately activate the dorsal stream by utilizing stimuli with a low spatial frequency, low contrast, and high temporal frequency (Kuba et al., 2007).

The transient/magnocellular system is considered to be faster than the parvocellular system and is engaged in exogenous attention processing (Steinman et al., 1997; Abrams and Christ, 2003; Laycock et al., 2008) [although not exclusively (Ries and Hopfinger, 2011)] and therefore might be more suitable for vMMN examination.

Because of selective deficits within the previously mentioned streams in some neuro-ophthalmic disorders, such as open angle glaucoma, multiple sclerosis, neuroborreliosis, amblyopia, among others (Kubova et al., 1996; Arakawa et al., 1999; Szanyi et al., 2012), the examination of the vMMN along the magnocellular pathway/dorsal stream might bring new information.

In our previous study, we used a paradigm for vMMN generation through the activation of the magnocellular pathway that met the requirements for refractoriness elimination (Kremlacek et al., 2006). For the experiment described in this study, we modified our previous design. We used radial motion (Kremlacek et al., 2004) for more effective standard/deviant peripheral activation and we applied an interleaved numeric task of different stimulus dimension for the control of attention. The interleaved design shortened the examination time and the use of numbers in the center of the visual field allowed for additional manipulations with attentional involvement.

The aim of this study was to evaluate the effect of task difficulty on an electrophysiological correlate of the violation of probability-based regularity, induced by the activation of magnocellular input via a motion sequence. We also sought to determine a sufficient level of task difficulty to allow for unbiased vMMN examination during clinical use.

## Methods

### Subjects

We examined a group of twelve healthy adult subjects (aged 21–61 years, 3 females) with no ophthalmologic or neurological abnormalities and with normal or corrected-to-normal visual acuity. Informed consent was obtained from each subject after they received an explanation of the test procedure. The study was approved by the Ethical Committee of the Faculty of Medicine in Hradec Kralove and experiments were conducted in accordance with the Declaration of Helsinki (World Medical Association, 2004).

### Stimuli

The stimulus consisted of a low contrast (10%) sinusoidal circular pattern outside of the central 10° of the visual field of 36 × 47°. The spatial frequency of the pattern decreased toward the periphery, from 0.4 to 0.2 c/°. The pattern changed every 200 ms in a sequence of expansion (100 ms) and contraction (100 ms) or in the opposite sequence (contraction followed by expansion), with a velocity from 12.5 to 25°/s, to keep the temporal frequency of 5 Hz constant within the stimulus field.

In between the motion sequences, the pattern was stationary for 600 ms. During this stationary phase, the fixation point in the center of the stimulus field was changed to a randomly selected digit from 1 to 8 for 200 ms.

**Figure 1:**
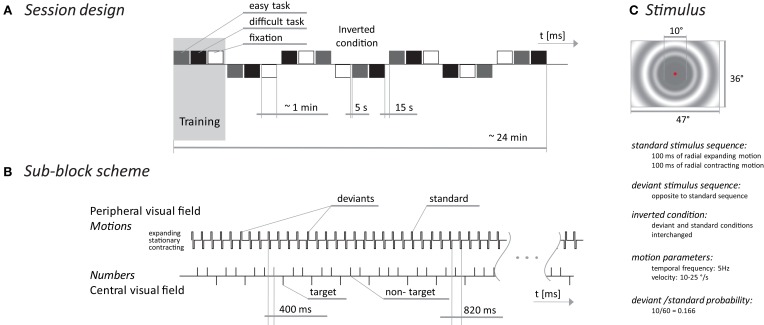
**MMN experimental scheme.** The session design **(A)** presents blocks (triplets) of sub-blocks with three different tasks. The sub-block scheme **(B)** shows a temporal diagram of events occurring in the peripheral part of the visual field (upper time line) and events in the central part of the screen during the oddball task. The stimulus **(C)** depicts the spatial/temporal properties of the peripheral stimuli.

The vMMN was elicited by a change in the sequence of the expanding/contracting radial motions while the subject visually fixated on the central part of the display. The ratio between deviant and standard stimuli was 0.17. In half of the recorded blocks, the standard stimulus was an expanding/contracting motion and the deviant was a contracting/expanding motion. During the second half of the blocks, the stimuli were interchanged (see Figure 1).

To explore the relationship between the vMMN and the amount of attention allocated outside the standard/deviant stimuli, we used three tasks: a simple central fixation requiring no overt behavioral response and an oddball task of two difficulties. During the oddball task, subjects were instructed to press a handheld button as soon as the number 1 (easy task) or the numbers 1, 4, or 8 (difficult task) appeared. The target to non-target ratio was 0.30 for both the difficult and easy tasks. The number of target stimuli was the same in both oddball tasks and it was twice the number of deviant stimuli.

The entire session consisted of 7 blocks and each block included three tasks that were presented pseudo-randomly in three sub-blocks, each lasting one minute. Stimulus presentation in each block was terminated when 10 deviant and 20 target stimuli were delivered. The number of standard and non-target stimuli was different in each block but corresponded with the previously mentioned probabilities. Between sub-blocks there were 5 s breaks and between blocks there were 15 s breaks with short joke texts presented on the screen to keep the subjects alert. The first block was used to familiarize the subjects with the tasks. The experiment timing and stimulus appearance are depicted in Figure 1. The stimuli were presented on a 21-inch computer monitor (Mitsubishi Diamond Pro 2070 SB, Japan). The monitor was driven using PsychToolbox (Brainard, 1997) at a 100 Hz. A mean screen luminance of 21 cd/m^2^ was used for all stimuli.

### Recording

vMMN acquisition was performed in a darkened, sound attenuated, electromagnetically shielded room, with a background luminance of 1 cd/m^2^. The subjects were seated and instructed to fixate on the center of the stimulus field.

Responses were recorded from 68 unipolar electrodes, including four EOG electrodes. The right earlobe (A2) served as a reference. The signal amplifier had a bandwidth of 0.3–100 Hz (Alien technik s.r.o., Czech Republic). The EEG was sampled at a rate of 1024 Hz and saved for off-line processing.

### Analysis

The data were processed using EEGlab (Delorme et al., 2011) and custom routines in Matlab release 2013a (Mathworks, USA). The recorded EEG was digitally band pass filtered (0.5–30.0 Hz) and divided into epochs of −99 to 400 ms in duration with respect to the onset of a standard/deviant stimulus. The baseline was defined as the mean amplitude in the period from −99 to 0 ms (prestimulus part) for each epoch. Epochs with amplitudes outside the range of ±50 μV were rejected (18% of all epochs). Channels with artifacts were removed and substituted by spatially interpolating the signal using EEGlab. Using this method, we interpolated one channel in 6 subjects, two channels in 3 subjects and three channels in one subject. To create session as short as possible, every second target was presented immediately after a deviant stimulus what systematically contaminated the responses to deviant stimuli and in lesser extend to the standard stimuli by the readiness potential (Bereitschafts Potential). The linear trend of in the epochs was removed to eliminate bias caused by the preparation (expectation) of responding to the oddball task. In each subject, we evaluated responses to the standard stimuli immediately preceding responses to the deviant stimuli (6 × 3 × 10 epochs). The responses to direct and “inverted” stimuli were pooled for the analysis.

The period containing a possible vMMN was identified as the local maxima of the global mean field power of the deviant—standard ERPs aggregated across subjects, task and blocks. Statistical analysis was performed on the mean amplitudes from the selected periods in the fronto-central and parietal regions, which were selected according to the vMMN distribution (see Figure 3).

A general linear model for repeated measures was applied to the mean amplitude with a three-factor design: condition (standard and deviant), region (fronto-central and parietal), and task (fixation only, easy and difficult task). The results are reported as statistically significant if *p* < 0.05.

The correlation between age and visually evoked potentials (Kuba et al., 2012) suggests that age might be used as a covariate in our analysis. We examined the correlation between age and the vMMN, but there was no significant correlation; therefore, only within subject factors without age as a covariate were used in the general linear model.

## Results

### Behavioral analysis

The reaction time for the easy task was 343 ± 46 ms, while for the difficult task subjects responded 392 ± 51 ms after the target number. The reaction times for the easy task were significantly shorter [paired *t*-test *t*_(9)_ = 5.8, *p* < 0.001]. Due to response box error, three subjects were excluded from the reaction time analysis.

### Electrophysiological data

**Figure 2:**
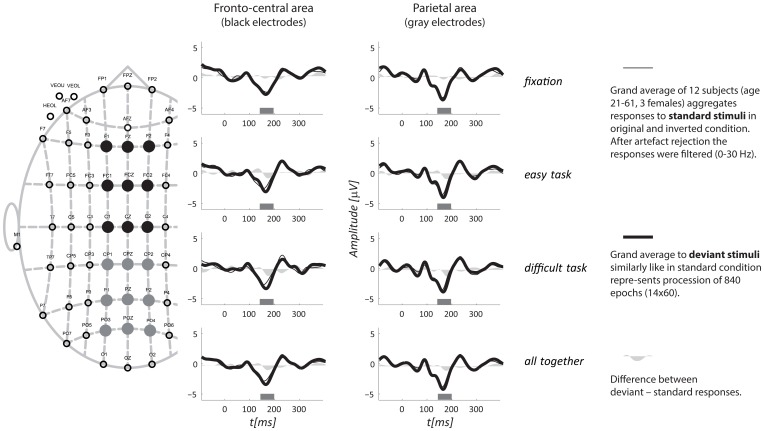
**Grand average ERPs for all three tasks aggregated from two regions.** A schematic layout of the recording electrodes with indication of the fronto-central (full black circles) and the parietal (full gray circles) regions of interest is in the left portion of the figure. The top three rows display responses from the three tasks separately, and the fourth row shows all tasks together. The interval of interest, for which the mean amplitude was evaluated, is depicted as a gray rectangle along horizontal axis.

**Figure 3:**
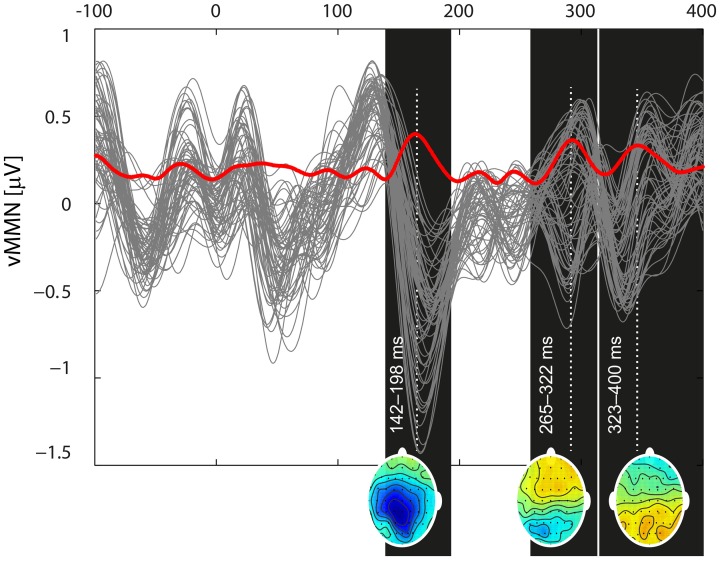
**Butterfly plot of the grand mean deviant-standard difference waveform at all channels including the vMMN (gray traces) and its global mean power (red trace) demonstrate the temporal dynamics of the vMMN.** Three local extremes around which the intervals of interest were selected (142–198, 265–332, and 324–400) are marked as black rectangles. For the time points indicated by a white dotted line, potential distributions are plotted at the bottom of the appropriate rectangles.

Based on the global mean field power of aggregated vMMN, three intervals were visually identified: 142–198, 265–322, and 323–400 ms (see Figure 3). The vMMN reached a maximum in two regions: the fronto-central (F_1_, F_Z_, F_2_, FC_1_, FC_Z_, FC_2_, C_1_, C_Z_, and C_2_) and the parietal regions (CP_1_, CP_Z_, CP_2_, P_1_, P_Z_, P_2_, PO_1_, PO_Z_, and PO_2_). The mean amplitude was evaluated in the aforementioned intervals and the regions of interest. The aggregated ERPs, together with the localization of electrodes, are depicted in Figure 2.

A general linear model for repeated measures was applied to the mean amplitudes with a three-factor design and showed a significant difference for only the first interval. The mean amplitudes are listed in Table 1. Statistical significance was reached for the factor of condition [*F*_(1, 11)_ = 17.40, *p* = 0.002] and for region [*F*_(1, 11)_ = 8.40, *p* = 0.014] but not for task [*F*_(2, 22)_ = 1.26, *p* = 0.30]. The analysis also indicated an interaction effect between task and amplitude in regions [*F*_(2, 22)_ = 4.16, *p* = 0.029], showing that the amplitudes in the fronto-central region decreased with the difficulty of the task, while they increased in the parietal area. This interaction did not occur with the standard/deviant condition; thus, it will not be further discussed. The other interactions did not reach statistical significance [condition × task *F*_(2, 22)_ = 0.66, *p* = 0.527; region × condition × task *F*_(2, 22)_ = 0.65, *p* = 0.534].

**Table 1:** **The table shows the mean amplitudes and standard deviations in the selected interval of 142–198 ms, from fronto-central and parietal derivations, for the standard and deviant conditions that were grouped together for the three different tasks**.

		**Mean amplitude ± SD [μV]; *n* = 12; 142–198 ms**	
**Task**	**Condition**	**Fronto-central a.**	**Centro-parietal a.**
Fixation	Standard	−2.74 ± 1.31	−2.16 ± 1.12
	Deviant	−2.96 ± 1.16	−2.38 ± 1.12
Easy	Standard	−2.32 ± 1.62	−1.75 ± 1.67
	Deviant	−2.98 ± 1.67	−2.16 ± 1.43
Difficult	Standard	−2.16 ± 1.71	−1.84 ± 1.54
	Deviant	−2.85 ± 1.21	−2.52 ± 1.17

The grand average ERPs, regions and the intervals of interest are depicted in Figure 2.

## Discussion

Our experiments have shown that the vMMN, evoked by a sequence of motions in periphery of the visual field, was not modulated by the difficulty of tasks that subjects solved in the central part of the visual field. A previous study by Pazo-Alvarez et al. (2004) used a similar design: a central task to control the attentional load and two moving gratings that appeared in the periphery and defined the standard/deviant condition by their direction of motion. They, in agreement with our results, did not find any effect of task difficulty on the generation of the vMMN. Our results are also similar to a study using a continuous performance task in the central part of the screen and standard/deviant stimuli presented as a grating in the periphery of the visual field (Heslenfeld, 2003). The authors did not report an effect of task difficulty on the vMMN found in the interval of 160–200 ms over the occipital, temporal or parietal areas.

However, our findings contradict several studies regarding the MMN in the auditory (for review see Sussman, 2007) and visual domains (Kimura et al., 2008; Czigler and Sulykos, 2010) where the attentional load or direction of attention modulated MMN. Such modulations are in agreement with the general effect of attention on the ERP (Luck et al., 2000). Some of these results do not directly contradict our results, such as the results for changes in the vMMN that were induced by the attention to a task, which were restricted to only interactions within the same stimulus dimension (i.e., the task was focused on color and the regularity was broken by a color change) (Czigler and Sulykos, 2010) while Heslenfeld's, Pazo-Alvarez's and our experiments violated regularity in different domain than tasks utilized. Another study (Kimura et al., 2008) presented deviant, standard and target stimuli in the same location, and therefore, overt attention was also orientated to the deviant stimulus. This limits direct comparisons with our results because, in our experiment, overt attention was located away from the standard/deviant stimuli.

There are also studies regarding brain metabolism with designs similar to ours. In an fMRI study, the perception of visual stimuli, such as optical flow, were modulated by the difficulty of an unrelated, spatially isolated task (Rees et al., 1997). Another similar study showed an effect of task difficulty on the perception of irrelevant color deviants (Yucel et al., 2007). These findings, unlike our findings and other electrophysiological studies (Heslenfeld, 2003; Pazo-Alvarez et al., 2004), may be attributed to using a different technique. ERP reflects transient, phase-locked events related to neural activity, whereas the blood oxygen level-dependent signal corresponds to sustained metabolic activity. It is possible to use an event-related fMRI design, but this approach cannot differentiate among processes occurring on a millisecond time scale. This discrepancy between electrophysiological and metabolic studies might be addressed in an experiment recording simultaneously EEG and fMRI.

Our results also contradict the “load theory” (Lavie et al., 2004), which states that the perception of a distractor depends on the task load and that the distractor is perceived when there are available attentional resources. Our results show that the distractors, for instance, standard and deviant stimuli, were processed by the sensory cortex, but there was no modulation of the response by task difficulty. One explanation might be that the tasks were so demanding that they exhausted all attentional resources. However, this seems unlikely because one of the tasks only required fixation on the center of the screen. Another possibility is that the tasks were insufficiently difficult, such that the attentional resources were altered so negligibly that the vMMN was not modulated. This is also unlikely because, in response to the deviant stimuli, there should be an attentional shift in the 200–300 ms interval (Heslenfeld et al., 1997) or at a later time point in a P3a component (Squires et al., 1975). We did not detect these components, and our results did not show an effect of task *per se*, nor its interaction with the condition factor (the standard/deviant stimuli).

Thus, we speculate that our experimental design presented so many transient changes (approximately 8/s—motion-onset, motion-reversal, motion-offset, pattern-on, and pattern-off, all happened within 600 ms; see Figure 1) that the standard/deviant difference was not salient enough to systematically capture subjects' attention despite the generation of the electrophysiological correlate in the vMMN. Some of the subjects were questioned after the experiment and they reported a lack of awareness of the peripheral regularity violation. Unfortunately, we do not have behavioral responses from all subjects; however, the data suggest that the attentional involvement in the peripheral stimuli was low.

The observation that the vMMN generated in our design did not change with task difficulty might be useful because it is desirable to dissociate the effect attentional bias from the genuine vMMN.

One of the goals of this study was to verify that the described protocol was suitable for a fast and reliable examination of the vMMN. In addition of the ability to elicit the vMMN, we found the following advantages of our design: (a) the sequence of motion in two directions avoided the possibility of refractoriness within the dorsal stream because the durations of the expanding and contracting motions within the single stimulus were equal; (b) the deviant stimuli did not elicit systematic changes or shifts in attention; (c) the responses to irrelevant stimuli were independent of central task difficulty; and (d) the radial motion avoids optokinetically induced eye movements.

However, this design has the following disadvantages: (a) we recorded small vMMN amplitudes, which makes the clinical use of this design difficult; and (b) the sequence had numerous target events that contaminated the responses to the irrelevant stimuli with slow readiness potentials, which subsequently had to be removed.

### Conflict of interest statement

The authors declare that the research was conducted in the absence of any commercial or financial relationships that could be construed as a potential conflict of interest.

## Footnotes

^1^The role of attention in the generation of the MMN is complicated because the MMN was shown to depend on the manipulation of attention, mainly during the formation of the response to standard stimuli (e.g., building a memory trace) (Sussman et al., 2002). When subjects ignored a regular pattern of oddball design, the MMN was generated as the result of sensory discrimination; however, when they were instructed to pay attention to the pattern in the same oddball sequence, the MMN diminished (Sussman et al., 2002). Currently, it is accepted that perceptual learning, which is a necessary process in MMN generation, can be influenced by attention (Sussman, 2007); however, the process should be unintentional (Kimura, 2012).
